# An In vitro Comparison of Furcal Perforation Repaired with Pro-root MTA and New Endodontic Cement in Primary Molar Teeth- A Microleakage Study

**Published:** 2014-03

**Authors:** R. Haghgoo, M. Niyakan, K. Nazari Moghaddam, S. Asgary, N. Mostafaloo

**Affiliations:** a Dept. of Pediatric Dentistry, Dental School, Shahed University, Tehran, Iran; b Dept. of Microbiology, Medical School, Shahed University, Tehran, Iran; c Dept. of Endodontics, Dental School, Shahed University, Tehran, Iran; d Dept. of Endodontic, Iranian Center for Endodontic Research, School of Dentistry, Shaheed Beheshti Medical University, Tehran, Iran; e Dentist, Private office

**Keywords:** Pro- root MTA, NEC, Furcal perforation, Primary molar

## Abstract

**Statement of Problem: **One of the most challenging procedural accidents during pulpotomy of primary molars is furcal perforation. To prevent bacterial invasion, the perforation site should be sealed as soon as possible.

**Purpose:** The aim of the current study is to investigate the ability of the pro-root MTA and new endodontic cement (NEC) in repairing the furcation perforations of primary molar teeth.

**Materials and Method:** In this in vitro study, 42 extracted primary molars were selected. Their roots were sectioned horizontally and standard access cavity was prepared. The orifices and the root apices were sealed with two layers of resin composite.The samples were randomly assigned into 2 groups. 6 teeth were considered as the positive and the negative controls. In the experimental groups; perforation was made. In group 1 and 2, perforation site received pro-root MTA and NEC respectively. The teeth were covered by two layers of nail polish except for the external surface of the perforation site. The negative control group received no repairing material. All teeth were mounted and sterilized for 24 hours. Lower chambers were filled with sterilized Muller Hinton broth. Bacterial suspension of Enterococcus faecalis in 0.5 McFarland was prepared. The repaired site was then exposed to the bacterial suspension of Enterococcus faecalis every 3 days. All samples were inserted in an incubator at 37^o^C and 100% humidity. The turbidity of the samples was detected for a period of 30 days. Data were analyzed by Chi- square test.

**Results:** 44% of samples in Pro- root group, 50% of the samples in the NEC group showed contaminations during 30 days. There was no significant difference between these two groups (p= 0.799).

**Conclusion:** With limitations of this study, Pro- root MTA and NEC showed similar capability in sealing the furcal perforations of the primary molars.

## Introduction

Furcal perforation is a communication between root canals and periodontal ligaments through the pulp chamber [1-2]. It is considered as an undesirable accident which affects the prognosis of the treatment. Furcal perforation can be induced by different reasons, including rampant caries, resorption and the misdirection of the bur  in preparing the access cavity of the pulp chamber while all these causes may lead to an inflammatory response in periodontal tissues [3-4]. Any delay in repairing results in the bacterial contamination of the perforation site which consequently leads to the gingival down-growth of the epithelium into the perforation area, inflammation, bone resorption, necrosis and eventual loss of the tooth [5-6]. Primary molar teeth are involved in mastication, speaking and esthetics, therefore, keeping them till eruption of permanent teeth eruption is unavoidable [7]. The repairing of furcal perforation can be achieved by using non surgical or surgical approach and employing different materials such as: amalgam, IRM, Gutta-percha, Cavit, light- cured GI cement, resin composite, MTA super EBA, calcium hydroxide and NEC as reported in many studies in the literature [2, 5, 8-10].

MTA (mineral trioxide aggregate), introduced to endodontics by Torabinejad in 1993, is derived from Portland cement and has been implemented successfully in the repair of lateral root and furcal perforations, apex genesis and as a vital pulp capping agent. Moreover, it was utilized as an apical plug in one visit apexification and as a root-end filling material [11-13. MTA has many properties inducing: hard tissue formation adjacent to pulp, low toxicity, antibacterial effect and inducing cementogenesis [14-16].

A new endodontic cement (NEC), also named CEM, was introduced by Asgary et al. in 2006 [17]. NEC is a white hydrophilic powder containing tricalcium phosphate, calcium sulfate, calcium silicate, calcium hydroxide, and calcium oxide to improve the physical and chemical properties of this innovative material. NEC releases calcium and phosphate ions which promote hydroxyl apatite formation [18]. NEC, having a similar pH as MTA, exhibit more flow, shorter working time and less film thickness compared to the MTA. Different in vivo and in vitro studies showed desirable outcomes [17-19].

The aim of this study was to evaluate the sealing ability of the pro-root MTA and new endodontic cement (NEC) in repairing the primary molar furcation perforation, concerning the bacterial micro leakage. 

## Materials and Method

The study sample comprised of 42 recently extracted mandibular molars with at least 3mm intact coronal walls on three sides, non-fused well-developed roots and no previous treatment. These teeth were cleansed by being placed in a 5.25% solution of sodium hypochlorite (Chemin Chemical Co; Tehran, Iran) for 10 minutes and then were washed with tap water. The roots were sectioned at 2mm under the CEJ. Then, with a diamond fissure bur (#08, D&Z Co; Wies Baden, Germany), cavities with 2 mm depth were prepared at root ends. Access cavities were made using a #5 round bur in a high speed handpiece with constant coolant in the experimental group of 42 teeth. The orifices and root ends were etched with phosphoric acid 37% (Kimia acid gel; CHEME Dent Co, Tehran , Iran) , bounded by Tetric N-Bond (Ivoclar Vivadent Co; USA) and filled with light -cured flowable restorative resin composite (Dia dent Co; south Korea). Perforations were made in the center of the pulp chamber floor using a #012 round bur in a low speed hand piece. The perforation diameter was equal to the bur diameter. Teeth were then rinsed with water and dried with oil-free air. A moist cotton pellet was placed between roots in the furcation area and the teeth were kept in an incubator at 37 ^o^ c for 24 hours. Six teeth were considered as the positive and the negative control groups. In all groups, except negative control groups, the perforation was performed by a #012 bur (D&Z Co; Wies Baden, Germany) at the center of the floor. Therefore, teeth were randomly assigned into two experimental groups.The CEM and Pro-Root MTA were both prepared according to the manufacturer’s instructions. In group 1 and 2, perforation site received Pro-root MTA (DENTSPLY Tulsa Dental Co; USA) and New endodontic cement (Bionque Dent; Yektazist Dandan Co, Iran) respectively. The positive control group received no repairing material. All the materials were packed on the perforation site. During packing, teeth were inserted into a wet sponge, up to their cervical part, to simulate the oral environment. External surfaces of teeth were covered by two layers of nail polish except for the perforation site. In the negative control group, no perforation was made and surfaces were covered with two layers of nail polish. The roots were mounted between the two chamber apparatus according to an experimental setup described by Barthel et al. [20].The surrounding acquaintance of the holes and the teeth were sealed with cyanoacrylate glue and were sterilized by ethylene oxide gas for 24 hours. A bacterial suspension of Enterococcus faecalis (E-faecalis) in 0.5 McFarland, was prepared and added to the upper chamber. To minimize the possibility of contamination, the lower chamber was filled with a sterile and clear Muller Hinton broth. Then the repaired furcation site was exposed to the bacterial suspension of E-faecalis every 3 days. All samples were inserted in an incubator at 37^o^C and 100% humidity to detect the turbidity of the samples for a period of 30 days. Data were recorded and were analyzed by Chi- square test.

## Results

The negative control group, in contrast to the positive control group, showed no turbidity during the experimental period. Eight (44%) samples (out of 18 total samples) in Pro-root group, nine (50%) samples (out of total 18 samples) in the NEC group, showed contamination during 30 days (Table1). There was no significant difference between two groups (p= 0.799).

**Table 1:** Microbial contamination with Pro-Root MTA and NEC

**Material**	**With leakage**	**Without leakage**	**Total**
Pro-Root MTA	8(44%)	10(56%)	18
NEC	9(50%)	9(%50)	18
Total	17	19	38

## Discussion

Furcal perforation is a procedural accident ; to prevent the consequent bacterial contamination the perforation sites should be repaired as quickly as possible by employing a biocompatible material [21].

The perforation site could be repaired surgically or non-surgically. The perforation, definitely, affects the outcome of the treatment and consequently the prognosis of the tooth. In other words, the main difficulty with non-surgical repair, even in case of proper sealing, is the extrusion of the repairing material into the periodontal ligament tissue [22]. In particular, this extrusion happens when the size of the perforation is large; which might interfere with periodontal ligament re-attachment [22].

Microleakage dye- study designs meet challenges. The dispute is because of the differences in the molecular size, surface tension and viscosity that possibly affect the penetration capability and the detection of the penetrating material [23-24]. Therefore, we employed bacterial growth in our microleakage study.

Microorganisms mostly cause pulp-periapical diseases, either directly or indirectly. Enterococcus faecalis is a gram-positive, facultative anaerobic bacterium which is commonly found in the previously root-filled teeth exhibiting persistent periapical lesions [25-26]. Enterococcus faecalis is the most prevalent microorganism that has been detected in 77% of the cases of failed endodontic therapy [27] and it seems that it is the most frequently present microorganisms in the root canals with failed endodontic treatments [28]. E-faecalis is a microorganism which is commonly detected in the asymptomatic persistent endodontic infections [29].

Its ability to invade the dentinal tubules, living without sufficient nutrition and even withstanding a lethal agent such as calcium hydroxide (Ca(OH)_2_), identifies it as a persisting endured endodontic pathogen [30].

To simulate the oral environment, we put wet cotton beneath the furcation site during the experimental period. Although a previous study showed that in the MTA-repaired furcal perforations, exposures to the normal saline, blood and lidocaine had no significant effect on the MTA properties. It was presumed that MTA would absorb sufficient moisture for its setting from the PDL tissue [31].

When freshly used at perforation site,  MTA and NEC showed some cytotoxicity which dissipated with time. From the clinical point of view, the furcation site in primary molar teeth, is thin and has many furcational canals which makes it more difficult to seal when perforation occurs. To repair perforation site, especially in primary molars, many attempts should be made to prevent imposing excessive pressure.

Our study revealed that there was no significant difference between MTA and NEC, regarding the bacterial microleakage; therefore, both materials can be used. NEC with shorter setting time and cheaper in price, can be a substitute for MTA. This finding is in accordance with the results yielded by Samiee et al. study [32].

Based on the results of our study, Pro- root MTA and NEC have similar effects in repairing of furcal perforation in primary molar teeth. Haghgoo and Abbasi in their study showed that root MTA and Pro-root can seal furcal perforations of primary molars [33]. This study evaluated the furcal perforation repair in the primary molars; however, our study employed a microleakage study model.

Haghgoo et al. in a study found that CEM and Proroot MTA have sealing ability of furcal perforation of primary molars [34]. This funding is in accordance with our study, however, we studied sealing ability of Root MTA and ProRoot MTA.

The popularity of NEC can be attributed to its low cytotoxicity on different cell strains [35-36]; which assists in promoting the osteogenesis and cementogenesis [15]. Moreover, NEC releases phosphorus and calcium ions from indigenous sources which provide a rich pool of OH^−^, Ca^2+^ and PO4^−^ ions [37-38]. These elements are used in the process of hydroxyapatite (HA) production.

## Conclusion

Based on the results of this study; both Pro-root MTA and NEC can be used for sealing the furcal perforations of the primary molar teeth.
